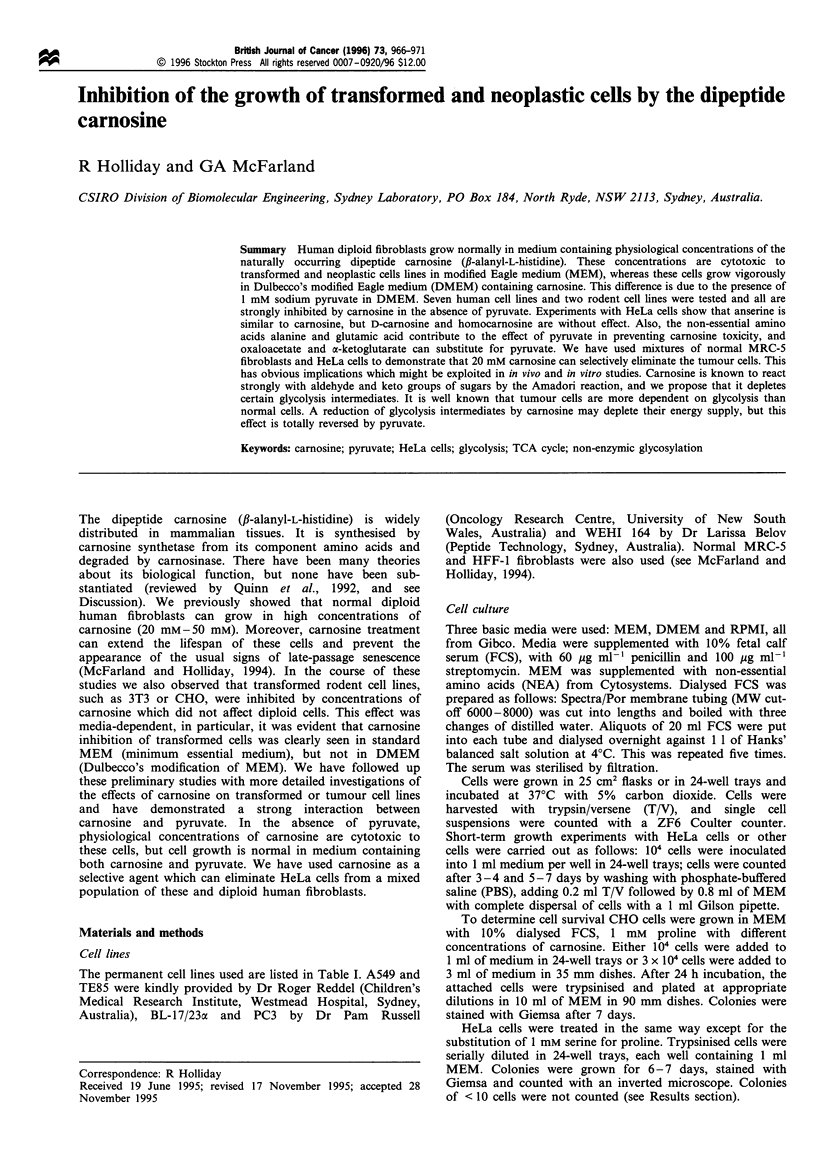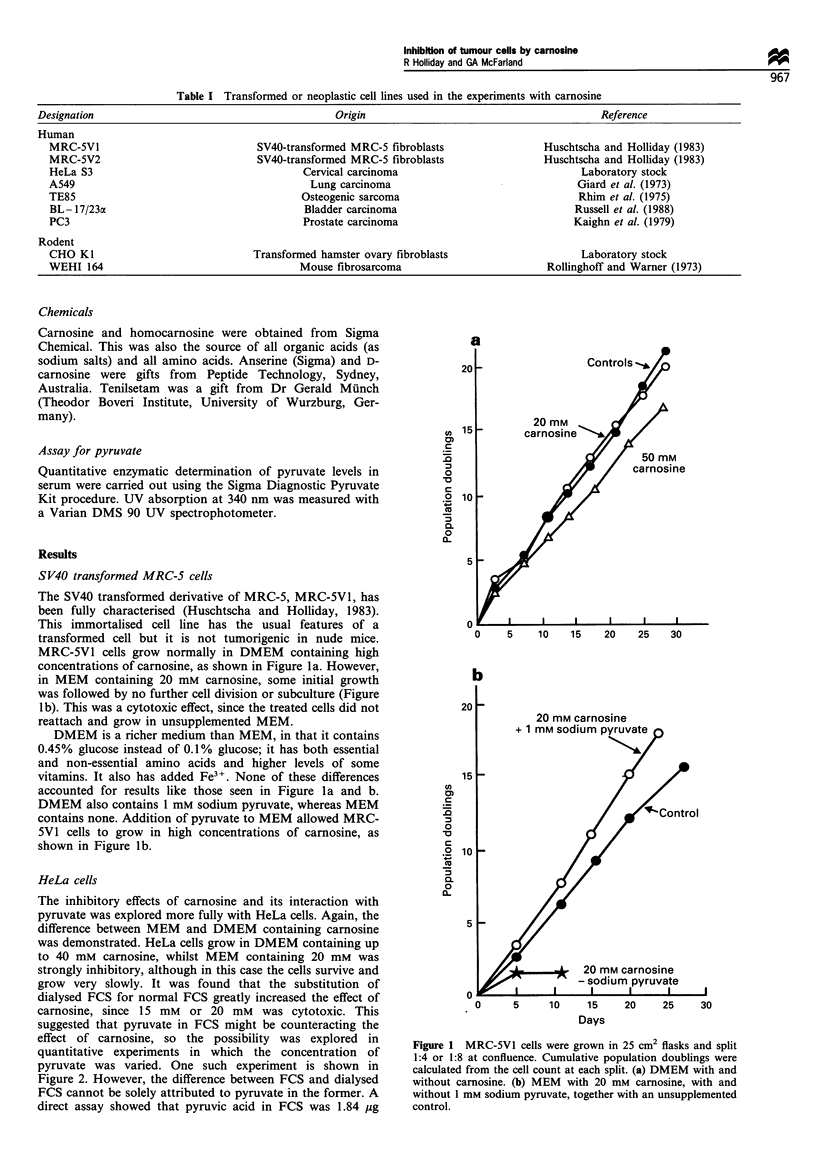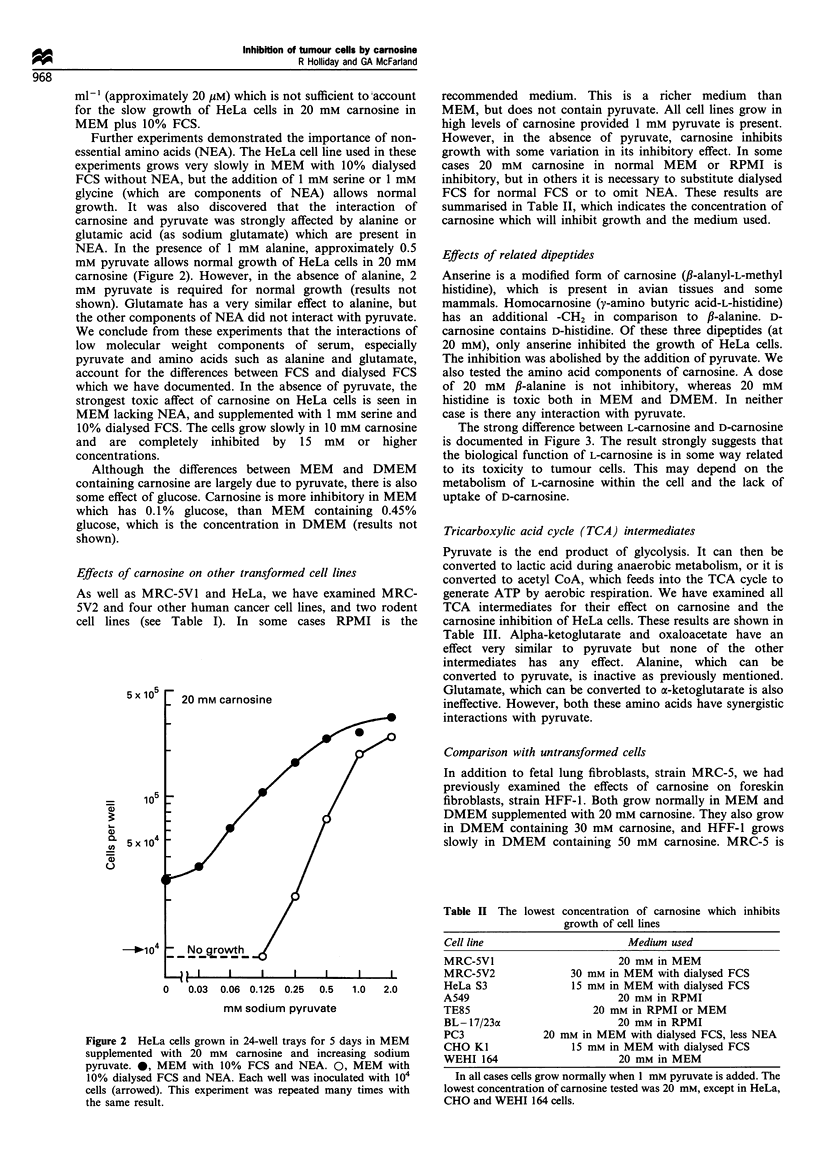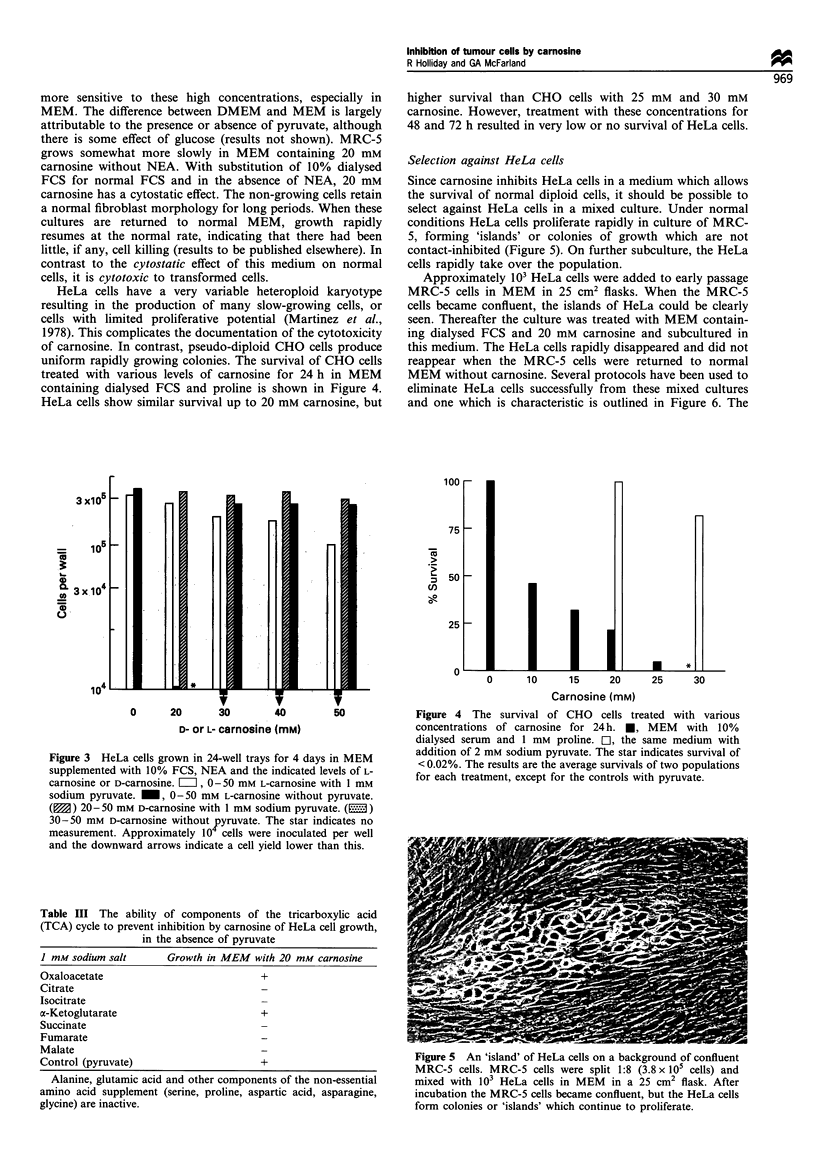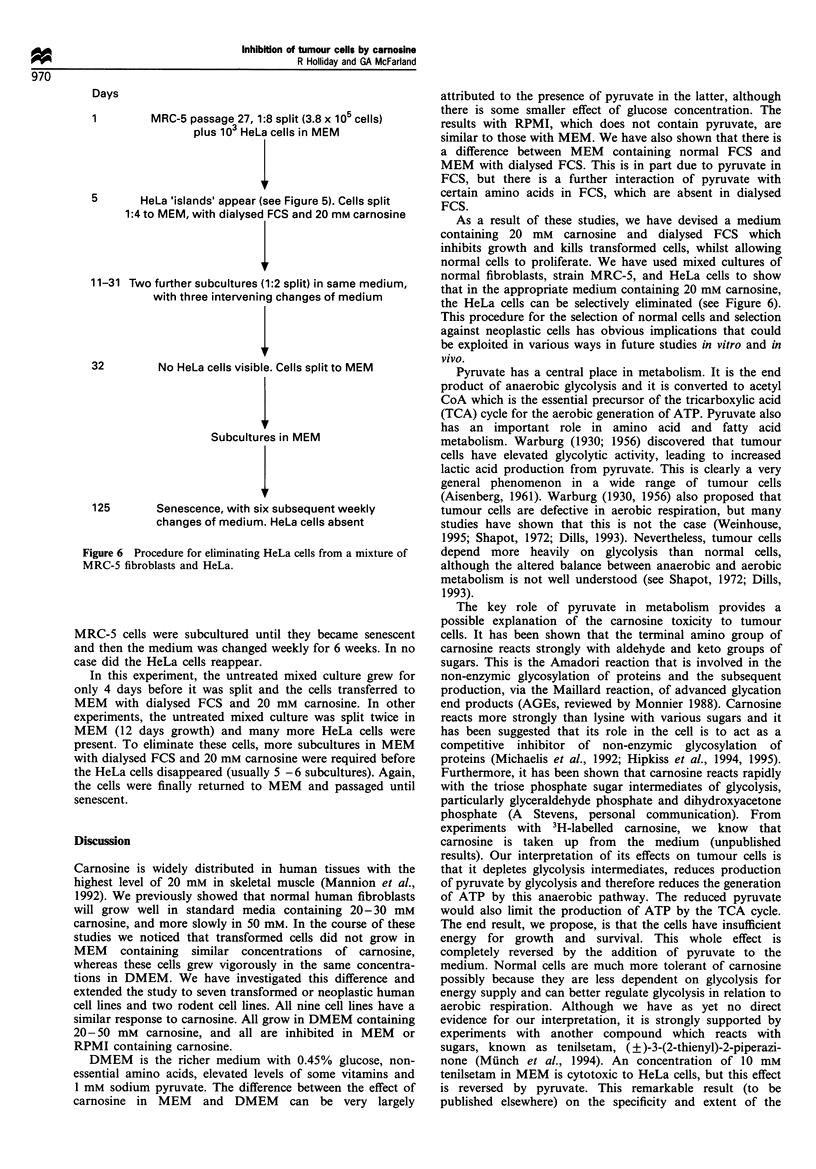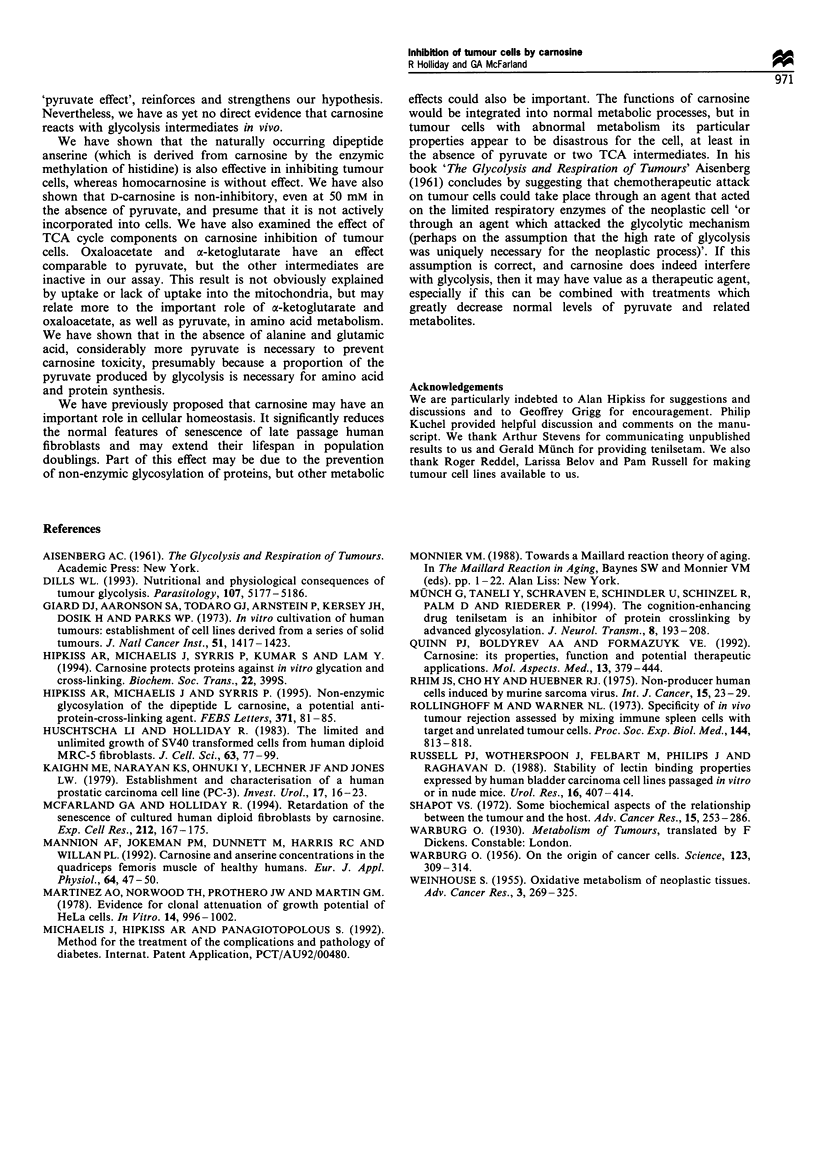# Inhibition of the growth of transformed and neoplastic cells by the dipeptide carnosine.

**DOI:** 10.1038/bjc.1996.189

**Published:** 1996-04

**Authors:** R. Holliday, G. A. McFarland

**Affiliations:** CSIRO Division of Biomolecular Engineering, Sydney Laboratory, NSW, Australia.

## Abstract

**Images:**


British Journal of Cancer (1996) 73, 966–971
© 1996 Stockton Press All rights reserved 0007–0920/96 $12.00

### Inhibition of the growth of transformed and neoplastic cells by the dipeptide carnosine

R Holliday and GA McFarland

*CSIRO Division of Biomolecular Engineering, Sydney Laboratory, PO Box 184, North Ryde, NSW 2113, Sydney, Australia.*

**Summary** Human diploid fibroblasts grow normally in medium containing physiological concentrations of the naturally occurring dipeptide carnosine (β-alanyl-L-histidine). These concentrations are cytotoxic to transformed and neoplastic cells lines in modified Eagle medium (MEM), whereas these cells grow vigorously in Dulbecco's modified Eagle medium (DMEM) containing carnosine. This difference is due to the presence of 1 mM sodium pyruvate in DMEM. Seven human cell lines and two rodent cell lines were tested and all are strongly inhibited by carnosine in the absence of pyruvate. Experiments with HeLa cells show that anserine is similar to carnosine, but D-carnosine and homocarnosine are without effect. Also, the non-essential amino acids alanine and glutamic acid contribute to the effect of pyruvate in preventing carnosine toxicity, and oxaloacetate and α-ketoglutarate can substitute for pyruvate. We have used mixtures of normal MRC-5 fibroblasts and HeLa cells to demonstrate that 20 mM carnosine can selectively eliminate the tumour cells. This has obvious implications which might be exploited in *in vivo* and *in vitro* studies. Carnosine is known to react strongly with aldehyde and keto groups of sugars by the Amadori reaction, and we propose that it depletes certain glycolysis intermediates. It is well known that tumour cells are more dependent on glycolysis than normal cells. A reduction of glycolysis intermediates by carnosine may deplete their energy supply, but this effect is totally reversed by pyruvate.

**Keywords:** carnosine; pyruvate; HeLa cells; glycolysis; TCA cycle; non-enzymic glycosylation

The dipeptide carnosine (β-alanyl-L-histidine) is widely distributed in mammalian tissues. It is synthesised by carnosine synthetase from its component amino acids and degraded by carnosinase. There have been many theories about its biological function, but none have been substantiated (reviewed by Quinn *et al.*, 1992, and see Discussion). We previously showed that normal diploid human fibroblasts can grow in high concentrations of carnosine (20 mM–50 mM). Moreover, carnosine treatment can extend the lifespan of these cells and prevent the appearance of the usual signs of late-passage senescence (McFarland and Holliday, 1994). In the course of these studies we also observed that transformed rodent cell lines, such as 3T3 or CHO, were inhibited by concentrations of carnosine which did not affect diploid cells. This effect was media-dependent, in particular, it was evident that carnosine inhibition of transformed cells was clearly seen in standard MEM (minimum essential medium), but not in DMEM (Dulbecco's modification of MEM). We have followed up these preliminary studies with more detailed investigations of the effects of carnosine on transformed or tumour cell lines and have demonstrated a strong interaction between carnosine and pyruvate. In the absence of pyruvate, physiological concentrations of carnosine are cytotoxic to these cells, but cell growth is normal in medium containing both carnosine and pyruvate. We have used carnosine as a selective agent which can eliminate HeLa cells from a mixed population of these and diploid human fibroblasts.

### Materials and methods

### *Cell lines*

The permanent cell lines used are listed in Table I. A549 and TE85 were kindly provided by Dr Roger Reddel (Children's Medical Research Institute, Westmead Hospital, Sydney, Australia), BL-17/23α and PC3 by Dr Pam Russell (Oncology Research Centre, University of New South Wales, Australia) and WEHI 164 by Dr Larissa Belov (Peptide Technology, Sydney, Australia). Normal MRC-5 and HFF-1 fibroblasts were also used (see McFarland and Holliday, 1994).

### *Cell culture*

Three basic media were used: MEM, DMEM and RPMI, all from Gibco. Media were supplemented with 10% fetal calf serum (FCS), with 60 $\mu g\ ml^{-1}$ penicillin and 100 $\mu g\ ml^{-1}$ streptomycin. MEM was supplemented with non-essential amino acids (NEA) from Cytosystems. Dialysed FCS was prepared as follows: Spectra/Por membrane tubing (MW cut-off 6000–8000) was cut into lengths and boiled with three changes of distilled water. Aliquots of 20 ml FCS were put into each tube and dialysed overnight against 1 l of Hanks' balanced salt solution at 4°C. This was repeated five times. The serum was sterilised by filtration.

Cells were grown in 25 $cm^2$ flasks or in 24-well trays and incubated at 37°C with 5% carbon dioxide. Cells were harvested with trypsin/versene (T/V), and single cell suspensions were counted with a ZF6 Coulter counter. Short-term growth experiments with HeLa cells or other cells were carried out as follows: $10^4$ cells were inoculated into 1 ml medium per well in 24-well trays; cells were counted after 3–4 and 5–7 days by washing with phosphate-buffered saline (PBS), adding 0.2 ml T/V followed by 0.8 ml of MEM with complete dispersal of cells with a 1 ml Gilson pipette.

To determine cell survival CHO cells were grown in MEM with 10% dialysed FCS, 1 mM proline with different concentrations of carnosine. Either $10^4$ cells were added to 1 ml of medium in 24-well trays or $3 \times 10^4$ cells were added to 3 ml of medium in 35 mm dishes. After 24 h incubation, the attached cells were trypsinised and plated at appropriate dilutions in 10 ml of MEM in 90 mm dishes. Colonies were stained with Giemsa after 7 days.

HeLa cells were treated in the same way except for the substitution of 1 mM serine for proline. Trypsinised cells were serially diluted in 24-well trays, each well containing 1 ml MEM. Colonies were grown for 6–7 days, stained with Giemsa and counted with an inverted microscope. Colonies of < 10 cells were not counted (see Results section).

Correspondence: R Holliday
Received 19 June 1995; revised 17 November 1995; accepted 28 November 1995

**Table I** Transformed or neoplastic cell lines used in the experiments with carnosine

| *Designation* | *Origin* | *Reference* |
|---|---|---|
| Human | | |
| MRC-5V1 | SV40-transformed MRC-5 fibroblasts | Huschtscha and Holliday (1983) |
| MRC-5V2 | SV40-transformed MRC-5 fibroblasts | Huschtscha and Holliday (1983) |
| HeLa S3 | Cervical carcinoma | Laboratory stock |
| A549 | Lung carcinoma | Giard *et al.* (1973) |
| TE85 | Osteogenic sarcoma | Rhim *et al.* (1975) |
| BL-17/23α | Bladder carcinoma | Russell *et al.* (1988) |
| PC3 | Prostate carcinoma | Kaighn *et al.* (1979) |
| Rodent | | |
| CHO K1 | Transformed hamster ovary fibroblasts | Laboratory stock |
| WEHI 164 | Mouse fibrosarcoma | Rollinghoff and Warner (1973) |

*Chemicals*

Carnosine and homocarnosine were obtained from Sigma Chemical. This was also the source of all organic acids (as sodium salts) and all amino acids. Anserine (Sigma) and D-carnosine were gifts from Peptide Technology, Sydney, Australia. Tenilsetam was a gift from Dr Gerald Münch (Theodor Boveri Institute, University of Wurzburg, Germany).

*Assay for pyruvate*

Quantitative enzymatic determination of pyruvate levels in serum were carried out using the Sigma Diagnostic Pyruvate Kit procedure. UV absorption at 340 nm was measured with a Varian DMS 90 UV spectrophotometer.

### Results

*SV40 transformed MRC-5 cells*

The SV40 transformed derivative of MRC-5, MRC-5V1, has been fully characterised (Huschtscha and Holliday, 1983). This immortalised cell line has the usual features of a transformed cell but it is not tumorigenic in nude mice. MRC-5V1 cells grow normally in DMEM containing high concentrations of carnosine, as shown in Figure 1a. However, in MEM containing 20 mM carnosine, some initial growth was followed by no further cell division or subculture (Figure 1b). This was a cytotoxic effect, since the treated cells did not reattach and grow in unsupplemented MEM.

DMEM is a richer medium than MEM, in that it contains 0.45% glucose instead of 0.1% glucose; it has both essential and non-essential amino acids and higher levels of some vitamins. It also has added $Fe^{3+}$. None of these differences accounted for results like those seen in Figure 1a and b. DMEM also contains 1 mM sodium pyruvate, whereas MEM contains none. Addition of pyruvate to MEM allowed MRC-5V1 cells to grow in high concentrations of carnosine, as shown in Figure 1b.

*HeLa cells*

The inhibitory effects of carnosine and its interaction with pyruvate was explored more fully with HeLa cells. Again, the difference between MEM and DMEM containing carnosine was demonstrated. HeLa cells grow in DMEM containing up to 40 mM carnosine, whilst MEM containing 20 mM was strongly inhibitory, although in this case the cells survive and grow very slowly. It was found that the substitution of dialysed FCS for normal FCS greatly increased the effect of carnosine, since 15 mM or 20 mM was cytotoxic. This suggested that pyruvate in FCS might be counteracting the effect of carnosine, so the possibility was explored in quantitative experiments in which the concentration of pyruvate was varied. One such experiment is shown in Figure 2. However, the difference between FCS and dialysed FCS cannot be solely attributed to pyruvate in the former. A direct assay showed that pyruvic acid in FCS was 1.84 μg

**Figure 1** MRC-5V1 cells were grown in 25 $cm^2$ flasks and split 1:4 or 1:8 at confluence. Cumulative population doublings were calculated from the cell count at each split. (**a**) DMEM with and without carnosine. (**b**) MEM with 20 mM carnosine, with and without 1 mM sodium pyruvate, together with an unsupplemented control.

ml$^{-1}$ (approximately 20 μM) which is not sufficient to account for the slow growth of HeLa cells in 20 mM carnosine in MEM plus 10% FCS.

Further experiments demonstrated the importance of non-essential amino acids (NEA). The HeLa cell line used in these experiments grows very slowly in MEM with 10% dialysed FCS without NEA, but the addition of 1 mM serine or 1 mM glycine (which are components of NEA) allows normal growth. It was also discovered that the interaction of carnosine and pyruvate was strongly affected by alanine or glutamic acid (as sodium glutamate) which are present in NEA. In the presence of 1 mM alanine, approximately 0.5 mM pyruvate allows normal growth of HeLa cells in 20 mM carnosine (Figure 2). However, in the absence of alanine, 2 mM pyruvate is required for normal growth (results not shown). Glutamate has a very similar effect to alanine, but the other components of NEA did not interact with pyruvate. We conclude from these experiments that the interactions of low molecular weight components of serum, especially pyruvate and amino acids such as alanine and glutamate, account for the differences between FCS and dialysed FCS which we have documented. In the absence of pyruvate, the strongest toxic affect of carnosine on HeLa cells is seen in MEM lacking NEA, and supplemented with 1 mM serine and 10% dialysed FCS. The cells grow slowly in 10 mM carnosine and are completely inhibited by 15 mM or higher concentrations.

Although the differences between MEM and DMEM containing carnosine are largely due to pyruvate, there is also some effect of glucose. Carnosine is more inhibitory in MEM which has 0.1% glucose, than MEM containing 0.45% glucose, which is the concentration in DMEM (results not shown).

### *Effects of carnosine on other transformed cell lines*

As well as MRC-5V1 and HeLa, we have examined MRC-5V2 and four other human cancer cell lines, and two rodent cell lines (see Table I). In some cases RPMI is the recommended medium. This is a richer medium than MEM, but does not contain pyruvate. All cell lines grow in high levels of carnosine provided 1 mM pyruvate is present. However, in the absence of pyruvate, carnosine inhibits growth with some variation in its inhibitory effect. In some cases 20 mM carnosine in normal MEM or RPMI is inhibitory, but in others it is necessary to substitute dialysed FCS for normal FCS or to omit NEA. These results are summarised in Table II, which indicates the concentration of carnosine which will inhibit growth and the medium used.

**Figure 2** HeLa cells grown in 24-well trays for 5 days in MEM supplemented with 20 mM carnosine and increasing sodium pyruvate. ●, MEM with 10% FCS and NEA. ○, MEM with 10% dialysed FCS and NEA. Each well was inoculated with $10^4$ cells (arrowed). This experiment was repeated many times with the same result.

### *Effects of related dipeptides*

Anserine is a modified form of carnosine (β-alanyl-L-methyl histidine), which is present in avian tissues and some mammals. Homocarnosine (γ-amino butyric acid-L-histidine) has an additional -$CH_2$ in comparison to β-alanine. D-carnosine contains D-histidine. Of these three dipeptides (at 20 mM), only anserine inhibited the growth of HeLa cells. The inhibition was abolished by the addition of pyruvate. We also tested the amino acid components of carnosine. A dose of 20 mM β-alanine is not inhibitory, whereas 20 mM histidine is toxic both in MEM and DMEM. In neither case is there any interaction with pyruvate.

The strong difference between L-carnosine and D-carnosine is documented in Figure 3. The result strongly suggests that the biological function of L-carnosine is in some way related to its toxicity to tumour cells. This may depend on the metabolism of L-carnosine within the cell and the lack of uptake of D-carnosine.

### *Tricarboxylic acid cycle (TCA) intermediates*

Pyruvate is the end product of glycolysis. It can then be converted to lactic acid during anaerobic metabolism, or it is converted to acetyl CoA, which feeds into the TCA cycle to generate ATP by aerobic respiration. We have examined all TCA intermediates for their effect on carnosine and the carnosine inhibition of HeLa cells. These results are shown in Table III. Alpha-ketoglutarate and oxaloacetate have an effect very similar to pyruvate but none of the other intermediates has any effect. Alanine, which can be converted to pyruvate, is inactive as previously mentioned. Glutamate, which can be converted to α-ketoglutarate is also ineffective. However, both these amino acids have synergistic interactions with pyruvate.

### *Comparison with untransformed cells*

In addition to fetal lung fibroblasts, strain MRC-5, we had previously examined the effects of carnosine on foreskin fibroblasts, strain HFF-1. Both grow normally in MEM and DMEM supplemented with 20 mM carnosine. They also grow in DMEM containing 30 mM carnosine, and HFF-1 grows slowly in DMEM containing 50 mM carnosine. MRC-5 is

**Table II** The lowest concentration of carnosine which inhibits growth of cell lines

| *Cell line* | *Medium used* |
|---|---|
| MRC-5V1 | 20 mM in MEM |
| MRC-5V2 | 30 mM in MEM with dialysed FCS |
| HeLa S3 | 15 mM in MEM with dialysed FCS |
| A549 | 20 mM in RPMI |
| TE85 | 20 mM in RPMI or MEM |
| BL-17/23α | 20 mM in RPMI |
| PC3 | 20 mM in MEM with dialysed FCS, less NEA |
| CHO K1 | 15 mM in MEM with dialysed FCS |
| WEHI 164 | 20 mM in MEM |

In all cases cells grow normally when 1 mM pyruvate is added. The lowest concentration of carnosine tested was 20 mM, except in HeLa, CHO and WEHI 164 cells.

more sensitive to these high concentrations, especially in MEM. The difference between DMEM and MEM is largely attributable to the presence or absence of pyruvate, although there is some effect of glucose (results not shown). MRC-5 grows somewhat more slowly in MEM containing 20 mM carnosine without NEA. With substitution of 10% dialysed FCS for normal FCS and in the absence of NEA, 20 mM carnosine has a cytostatic effect. The non-growing cells retain a normal fibroblast morphology for long periods. When these cultures are returned to normal MEM, growth rapidly resumes at the normal rate, indicating that there had been little, if any, cell killing (results to be published elsewhere). In contrast to the *cytostatic* effect of this medium on normal cells, it is *cytotoxic* to transformed cells.

HeLa cells have a very variable heteroploid karyotype resulting in the production of many slow-growing cells, or cells with limited proliferative potential (Martinez *et al.*, 1978). This complicates the documentation of the cytotoxicity of carnosine. In contrast, pseudo-diploid CHO cells produce uniform rapidly growing colonies. The survival of CHO cells treated with various levels of carnosine for 24 h in MEM containing dialysed FCS and proline is shown in Figure 4. HeLa cells show similar survival up to 20 mM carnosine, but higher survival than CHO cells with 25 mM and 30 mM carnosine. However, treatment with these concentrations for 48 and 72 h resulted in very low or no survival of HeLa cells.

*Selection against HeLa cells*

Since carnosine inhibits HeLa cells in a medium which allows the survival of normal diploid cells, it should be possible to select against HeLa cells in a mixed culture. Under normal conditions HeLa cells proliferate rapidly in culture of MRC-5, forming 'islands' or colonies of growth which are not contact-inhibited (Figure 5). On further subculture, the HeLa cells rapidly take over the population.

Approximately $10^3$ HeLa cells were added to early passage MRC-5 cells in MEM in 25 $cm^2$ flasks. When the MRC-5 cells became confluent, the islands of HeLa could be clearly seen. Thereafter the culture was treated with MEM containing dialysed FCS and 20 mM carnosine and subcultured in this medium. The HeLa cells rapidly disappeared and did not reappear when the MRC-5 cells were returned to normal MEM without carnosine. Several protocols have been used to eliminate HeLa cells successfully from these mixed cultures and one which is characteristic is outlined in Figure 6. The

**Figure 3** HeLa cells grown in 24-well trays for 4 days in MEM supplemented with 10% FCS, NEA and the indicated levels of L-carnosine or D-carnosine. □, 0–50 mM L-carnosine with 1 mM sodium pyruvate. ■, 0–50 mM L-carnosine without pyruvate. (▨) 20–50 mM D-carnosine with 1 mM sodium pyruvate. (▦) 30–50 mM D-carnosine without pyruvate. The star indicates no measurement. Approximately $10^4$ cells were inoculated per well and the downward arrows indicate a cell yield lower than this.

**Figure 4** The survival of CHO cells treated with various concentrations of carnosine for 24 h. ■, MEM with 10% dialysed serum and 1 mM proline. □, the same medium with addition of 2 mM sodium pyruvate. The star indicates survival of < 0.02%. The results are the average survivals of two populations for each treatment, except for the controls with pyruvate.

**Table III** The ability of components of the tricarboxylic acid (TCA) cycle to prevent inhibition by carnosine of HeLa cell growth, in the absence of pyruvate

| *1 mM sodium salt* | *Growth in MEM with 20 mM carnosine* |
|---|---|
| Oxaloacetate | + |
| Citrate | – |
| Isocitrate | – |
| α-Ketoglutarate | + |
| Succinate | – |
| Fumarate | – |
| Malate | – |
| Control (pyruvate) | + |

Alanine, glutamic acid and other components of the non-essential amino acid supplement (serine, proline, aspartic acid, asparagine, glycine) are inactive.

**Figure 5** An 'island' of HeLa cells on a background of confluent MRC-5 cells. MRC-5 cells were split 1:8 ($3.8 \times 10^5$ cells) and mixed with $10^3$ HeLa cells in MEM in a 25 $cm^2$ flask. After incubation the MRC-5 cells became confluent, but the HeLa cells form colonies or 'islands' which continue to proliferate.

**Figure 6** Procedure for eliminating HeLa cells from a mixture of MRC-5 fibroblasts and HeLa.

MRC-5 cells were subcultured until they became senescent and then the medium was changed weekly for 6 weeks. In no case did the HeLa cells reappear.

In this experiment, the untreated mixed culture grew for only 4 days before it was split and the cells transferred to MEM with dialysed FCS and 20 mM carnosine. In other experiments, the untreated mixed culture was split twice in MEM (12 days growth) and many more HeLa cells were present. To eliminate these cells, more subcultures in MEM with dialysed FCS and 20 mM carnosine were required before the HeLa cells disappeared (usually 5 – 6 subcultures). Again, the cells were finally returned to MEM and passaged until senescent.

### Discussion

Carnosine is widely distributed in human tissues with the highest level of 20 mM in skeletal muscle (Mannion *et al.*, 1992). We previously showed that normal human fibroblasts will grow well in standard media containing 20–30 mM carnosine, and more slowly in 50 mM. In the course of these studies we noticed that transformed cells did not grow in MEM containing similar concentrations of carnosine, whereas these cells grew vigorously in the same concentrations in DMEM. We have investigated this difference and extended the study to seven transformed or neoplastic human cell lines and two rodent cell lines. All nine cell lines have a similar response to carnosine. All grow in DMEM containing 20–50 mM carnosine, and all are inhibited in MEM or RPMI containing carnosine.

DMEM is the richer medium with 0.45% glucose, non-essential amino acids, elevated levels of some vitamins and 1 mM sodium pyruvate. The difference between the effect of carnosine in MEM and DMEM can be very largely attributed to the presence of pyruvate in the latter, although there is some smaller effect of glucose concentration. The results with RPMI, which does not contain pyruvate, are similar to those with MEM. We have also shown that there is a difference between MEM containing normal FCS and MEM with dialysed FCS. This is in part due to pyruvate in FCS, but there is a further interaction of pyruvate with certain amino acids in FCS, which are absent in dialysed FCS.

As a result of these studies, we have devised a medium containing 20 mM carnosine and dialysed FCS which inhibits growth and kills transformed cells, whilst allowing normal cells to proliferate. We have used mixed cultures of normal fibroblasts, strain MRC-5, and HeLa cells to show that in the appropriate medium containing 20 mM carnosine, the HeLa cells can be selectively eliminated (see Figure 6). This procedure for the selection of normal cells and selection against neoplastic cells has obvious implications that could be exploited in various ways in future studies *in vitro* and *in vivo*.

Pyruvate has a central place in metabolism. It is the end product of anaerobic glycolysis and it is converted to acetyl CoA which is the essential precursor of the tricarboxylic acid (TCA) cycle for the aerobic generation of ATP. Pyruvate also has an important role in amino acid and fatty acid metabolism. Warburg (1930; 1956) discovered that tumour cells have elevated glycolytic activity, leading to increased lactic acid production from pyruvate. This is clearly a very general phenomenon in a wide range of tumour cells (Aisenberg, 1961). Warburg (1930, 1956) also proposed that tumour cells are defective in aerobic respiration, but many studies have shown that this is not the case (Weinhouse, 1995; Shapot, 1972; Dills, 1993). Nevertheless, tumour cells depend more heavily on glycolysis than normal cells, although the altered balance between anaerobic and aerobic metabolism is not well understood (see Shapot, 1972; Dills, 1993).

The key role of pyruvate in metabolism provides a possible explanation of the carnosine toxicity to tumour cells. It has been shown that the terminal amino group of carnosine reacts strongly with aldehyde and keto groups of sugars. This is the Amadori reaction that is involved in the non-enzymic glycosylation of proteins and the subsequent production, via the Maillard reaction, of advanced glycation end products (AGEs, reviewed by Monnier 1988). Carnosine reacts more strongly than lysine with various sugars and it has been suggested that its role in the cell is to act as a competitive inhibitor of non-enzymic glycosylation of proteins (Michaelis *et al.*, 1992; Hipkiss *et al.*, 1994, 1995). Furthermore, it has been shown that carnosine reacts rapidly with the triose phosphate sugar intermediates of glycolysis, particularly glyceraldehyde phosphate and dihydroxyacetone phosphate (A Stevens, personal communication). From experiments with $^{3}H$-labelled carnosine, we know that carnosine is taken up from the medium (unpublished results). Our interpretation of its effects on tumour cells is that it depletes glycolysis intermediates, reduces production of pyruvate by glycolysis and therefore reduces the generation of ATP by this anaerobic pathway. The reduced pyruvate would also limit the production of ATP by the TCA cycle. The end result, we propose, is that the cells have insufficient energy for growth and survival. This whole effect is completely reversed by the addition of pyruvate to the medium. Normal cells are much more tolerant of carnosine possibly because they are less dependent on glycolysis for energy supply and can better regulate glycolysis in relation to aerobic respiration. Although we have as yet no direct evidence for our interpretation, it is strongly supported by experiments with another compound which reacts with sugars, known as tenilsetam, (±)-3-(2-thienyl)-2-piperazinone (Münch *et al.*, 1994). An concentration of 10 mM tenilsetam in MEM is cytotoxic to HeLa cells, but this effect is reversed by pyruvate. This remarkable result (to be published elsewhere) on the specificity and extent of the

'pyruvate effect', reinforces and strengthens our hypothesis. Nevertheless, we have as yet no direct evidence that carnosine reacts with glycolysis intermediates *in vivo*.

We have shown that the naturally occurring dipeptide anserine (which is derived from carnosine by the enzymic methylation of histidine) is also effective in inhibiting tumour cells, whereas homocarnosine is without effect. We have also shown that D-carnosine is non-inhibitory, even at 50 mM in the absence of pyruvate, and presume that it is not actively incorporated into cells. We have also examined the effect of TCA cycle components on carnosine inhibition of tumour cells. Oxaloacetate and α-ketoglutarate have an effect comparable to pyruvate, but the other intermediates are inactive in our assay. This result is not obviously explained by uptake or lack of uptake into the mitochondria, but may relate more to the important role of α-ketoglutarate and oxaloacetate, as well as pyruvate, in amino acid metabolism. We have shown that in the absence of alanine and glutamic acid, considerably more pyruvate is necessary to prevent carnosine toxicity, presumably because a proportion of the pyruvate produced by glycolysis is necessary for amino acid and protein synthesis.

We have previously proposed that carnosine may have an important role in cellular homeostasis. It significantly reduces the normal features of senescence of late passage human fibroblasts and may extend their lifespan in population doublings. Part of this effect may be due to the prevention of non-enzymic glycosylation of proteins, but other metabolic effects could also be important. The functions of carnosine would be integrated into normal metabolic processes, but in tumour cells with abnormal metabolism its particular properties appear to be disastrous for the cell, at least in the absence of pyruvate or two TCA intermediates. In his book '*The Glycolysis and Respiration of Tumours*' Aisenberg (1961) concludes by suggesting that chemotherapeutic attack on tumour cells could take place through an agent that acted on the limited respiratory enzymes of the neoplastic cell 'or through an agent which attacked the glycolytic mechanism (perhaps on the assumption that the high rate of glycolysis was uniquely necessary for the neoplastic process)'. If this assumption is correct, and carnosine does indeed interfere with glycolysis, then it may have value as a therapeutic agent, especially if this can be combined with treatments which greatly decrease normal levels of pyruvate and related metabolites.

**Acknowledgements**
We are particularly indebted to Alan Hipkiss for suggestions and discussions and to Geoffrey Grigg for encouragement. Philip Kuchel provided helpful discussion and comments on the manuscript. We thank Arthur Stevens for communicating unpublished results to us and Gerald Münch for providing tenilsetam. We also thank Roger Reddel, Larissa Belov and Pam Russell for making tumour cell lines available to us.

### References

AISENBERG AC. (1961). *The Glycolysis and Respiration of Tumours.* Academic Press: New York.

DILLS WL. (1993). Nutritional and physiological consequences of tumour glycolysis. *Parasitology*, **107**, 5177–5186.

GIARD DJ, AARONSON SA, TODARO GJ, ARNSTEIN P, KERSEY JH, DOSIK H AND PARKS WP. (1973). *In vitro* cultivation of human tumours: establishment of cell lines derived from a series of solid tumours. *J. Natl Cancer Inst.*, **51**, 1417–1423.

HIPKISS AR, MICHAELIS J, SYRRIS P, KUMAR S AND LAM Y. (1994). Carnosine protects proteins against *in vitro* glycation and cross-linking. *Biochem. Soc. Trans.*, **22**, 399S.

HIPKISS AR, MICHAELIS J AND SYRRIS P. (1995). Non-enzymic glycosylation of the dipeptide L carnosine, a potential anti-protein-cross-linking agent. *FEBS Letters*, **371**, 81–85.

HUSCHTSCHA LI AND HOLLIDAY R. (1983). The limited and unlimited growth of SV40 transformed cells from human diploid MRC-5 fibroblasts. *J. Cell. Sci.*, **63**, 77–99.

KAIGHN ME, NARAYAN KS, OHNUKI Y, LECHNER JF AND JONES LW. (1979). Establishment and characterisation of a human prostatic carcinoma cell line (PC-3). *Invest. Urol.*, **17**, 16–23.

MCFARLAND GA AND HOLLIDAY R. (1994). Retardation of the senescence of cultured human diploid fibroblasts by carnosine. *Exp. Cell Res.*, **212**, 167–175.

MANNION AF, JOKEMAN PM, DUNNETT M, HARRIS RC AND WILLAN PL. (1992). Carnosine and anserine concentrations in the quadriceps femoris muscle of healthy humans. *Eur. J. Appl. Physiol.*, **64**, 47–50.

MARTINEZ AO, NORWOOD TH, PROTHERO JW AND MARTIN GM. (1978). Evidence for clonal attenuation of growth potential of HeLa cells. *In Vitro*. **14**, 996–1002.

MICHAELIS J, HIPKISS AR AND PANAGIOTOPOLOUS S. (1992). Method for the treatment of the complications and pathology of diabetes. Internat. Patent Application, PCT/AU92/00480.

MONNIER VM. (1988). Towards a Maillard reaction theory of aging. In *The Maillard Reaction in Aging*, Baynes SW and Monnier VM (eds). pp. 1–22. Alan Liss: New York.

MÜNCH G, TANELI Y, SCHRAVEN E, SCHINDLER U, SCHINZEL R, PALM D AND RIEDERER P. (1994). The cognition-enhancing drug tenilsetam is an inhibitor of protein crosslinking by advanced glycosylation. *J. Neurol. Transm.*, **8**, 193–208.

QUINN PJ, BOLDYREV AA AND FORMAZUYK VE. (1992). Carnosine: its properties, function and potential therapeutic applications. *Mol. Aspects. Med.*, **13**, 379–444.

RHIM JS, CHO HY AND HUEBNER RJ. (1975). Non-producer human cells induced by murine sarcoma virus. *Int. J. Cancer*, **15**, 23–29.

ROLLINGHOFF M AND WARNER NL. (1973). Specificity of *in vivo* tumour rejection assessed by mixing immune spleen cells with target and unrelated tumour cells. *Proc. Soc. Exp. Biol. Med.*, **144**, 813–818.

RUSSELL PJ, WOTHERSPOON J, FELBART M, PHILIPS J AND RAGHAVAN D. (1988). Stability of lectin binding properties expressed by human bladder carcinoma cell lines passaged *in vitro* or in nude mice. *Urol. Res.*, **16**, 407–414.

SHAPOT VS. (1972). Some biochemical aspects of the relationship between the tumour and the host. *Adv. Cancer Res.*, **15**, 253–286.

WARBURG O. (1930). *Metabolism of Tumours*, translated by F Dickens. Constable: London.

WARBURG O. (1956). On the origin of cancer cells. *Science*, **123**, 309–314.

WEINHOUSE S. (1955). Oxidative metabolism of neoplastic tissues. *Adv. Cancer Res.*, **3**, 269–325.